# Whole genome sequencing in cats, identifies new models for blindness in *AIPL1* and somite segmentation in *HES7*

**DOI:** 10.1186/s12864-016-2595-4

**Published:** 2016-03-31

**Authors:** Leslie A. Lyons, Erica K. Creighton, Hasan Alhaddad, Holly C. Beale, Robert A. Grahn, HyungChul Rah, David J. Maggs, Christopher R. Helps, Barbara Gandolfi

**Affiliations:** Department of Veterinary Medicine and Surgery, College of Veterinary Medicine, University of Missouri - Columbia, E109 Vet Med Building, 1600 E. Rollins Street, Columbia, MO 65211 USA; College of Science, Kuwait University, Safat, 13060 Kuwait; Maverix Biomics, Inc., San Mateo, CA 94402 USA; Veterinary Genetics Laboratory, School of Veterinary Medicine, University of California - Davis, Davis, CA 95616 USA; Graduate School of Health Science Business Convergence, College of Medicine, Chungbuk National University, Chongju, Chungbuk Province 28644 South Korea; Department of Surgical and Radiological Sciences, School of Veterinary Medicine, University of California - Davis, Davis, CA 95616 USA; Langford Veterinary Services, University of Bristol, Langford, Bristol, BS40 5DU UK

**Keywords:** *Aryl-hydrocarbon-interacting receptor protein-like 1*, Domestic cat, *Felis silvestris catus*, *Hairy and Enhancer of Split 7*, LCA4, Progressive retinal atrophy

## Abstract

**Background:**

The reduced cost and improved efficiency of whole genome sequencing (WGS) is drastically improving the development of cats as biomedical models. Persian cats are models for Leber’s congenital amaurosis (LCA), the most severe and earliest onset form of visual impairment in humans. Cats with innocuous breed-defining traits, such as a bobbed tail, can also be models for somite segmentation and vertebral column development.

**Methods:**

The first WGS in cats was conducted on a trio segregating for LCA and the bobbed tail abnormality. Variants were identified using FreeBayes and effects predicted using SnpEff. Variants within a known haplotype block for cat LCA and specific candidate genes for both phenotypes were prioritized by the predicted variant effect on the proteins and concordant segregation within the trio. The efficiency of WGS of a single trio of domestic cats was evaluated.

**Results:**

A stop gain was identified at position c.577C > T in cat *AIPL1,* a predicted p.Arg193*. A c.5A > G variant causing a p.V2A was identified in *HES7*. The variants segregated concordantly in a Persian – Japanese bobtail pedigree. Over 1700 cats from 40 different breeds and populations were genotyped for the *AIPL1* variant, defining an allelic frequency in only Persian –related breeds of 1.15 %. A sub-set of cats was genotyped for the *HES7* variant, supporting the variant as private to the Japanese bobtail breed. Approximately 18 million SNPs were identified for application in cat research. The cat *AIPL1* variant would have been considered a high priority variant for evaluation, regardless of *a priori* knowledge from previous genetic studies.

**Conclusions:**

This study represents the first effort of the 99 Lives Cat Genome Sequencing Initiative to identify disease - causing variants in the domestic cat using WGS. The current cat reference assembly is efficient for gene and variant identification. However, as the feline variant database improves, development of cats as biomedical models for human disease will be more efficient, providing an alternative, large animal model for drug and gene therapy trials. Undiagnosed human patients with early-onset blindness should be screened for this *AIPL1* variant. The *HES7* variant should further calibrate the somite segmentation clock.

**Electronic supplementary material:**

The online version of this article (doi:10.1186/s12864-016-2595-4) contains supplementary material, which is available to authorized users.

## Background

Whole genome sequencing (WGS) is becoming the standard of health care in humans [1–9], and is commonly used to discover causal variants in children with otherwise undiagnosed congenital defects [10–13]. The 50-h and now the 26-h genome efforts have demonstrated how genome medicine can be applied to health management for acute care patients with time-critical morbidity and mortalities [10, 11]. The improved efficiency and lower costs of WGS now apply to all animal species, including pet cats. Over 80 million cats are owned in the USA and their roles as family-members and their health care are increasingly priorities for owners [14, 15]. Clinical trials of client-owned animals will advance both veterinary and human medicine [16]. Research colonies of cats have demonstrated their role as biomedical models for the development of gene and enzyme therapies [17, 18]. Indeed, gene therapy for canine Leber’s congenital amaurosis (LCA) has restored vision to the blind [19] and a variety of LCA clinical trials are underway in human patients [20–23].

Congenital amaurosis (MIM: 204000) was first described by Leber in the 1800’s [24, 25] and is now recognized as a heterogeneous group of early-onset childhood retinal dystrophies. LCA is classified as the most severe form of retinopathy [26]. LCA has a worldwide prevalence of 1 in 81,000 to 1 in 33,000 newborn babies, accounting for ≥ 5 % of all inherited retinopathies and approximately 20 % of children attending schools for the blind around the world [27–29]. Nineteen different genes are currently associated with the various forms of LCA [30, 31] (http://omim.org/phenotypicSeries/PS204000). Variants at an autosomal recessive locus, LCA4 (MIM: 604393), are caused by DNA variants in *AIPL1* (MIM: 604392) on human chromosome 17p13.1 [32–34]. *Aryl-hydrocarbon-interacting receptor protein-like 1* (*AIPL1)* variants cause approximately 7 % of LCA worldwide and may also cause dominant retinopathy [33, 34].

The Persian cat has been proposed as an animal model for retinal degeneration, specifically LCA [35–37]. Previous studies have localized the Persian cat progressive retinal atrophy to a 1.3 Mb region on cat chromosome E1, which is homologous to human chromosome 17 [35]. At the time of analysis, the publically available reference sequence, an Abyssinian cat, had over 11 Mb of genomic sequence in unplaced scaffolds [38]. The incomplete reference obstructed a full examination of an LCA gene, *AIPL1* within the haplotype block due to a gap in the cat genomic sequence.

WGS is feasible for domestic cats and is a highly efficient method to genetically examine several interesting phenotypes, in one cat, that do not have overlapping or compromising health effects. To maintain diversity in the Persian cat colony segregating for PRA, several innocuous phenotypes that are specific to pedigreed cats were introduced by outcrossing to cats of unrelated breeds. The bobbed tail of the Japanese bobtail cat breed results from abnormal caudal vertebrae morphology [39]. In addition, bobbed-tailed cats are typically lack a vertebra from the thoracic or lumbar regions, promoting this cat breed a model for spinal column development and somite segmentation [40–44]. Genes involved with somite segmentation are responsible for a severe disease in humans and dogs, spondylocostal dysostosis [45–47].

In this study, the Persian cat PRA is defined as a new model for LCA and the bobbed tail cat is a new model for spinal cord development. Using WGS of a trio of cats segregating for multiple traits, a gene within a ~1.3 Mb targeted region of interest from a previous genome-wide association study (GWAS) was implicated for Persian cat PRA. Without a priori knowledge of the GWAS targeted region, the LCA variant would have been identified based on proper segregation in the trio and the present of a stop gain variant in a known LCA gene. This feline PRA model is now available to the vision science community for gene therapies studies, and has potential to support both veterinary and human medicine. In addition, a gene associated with somite segmentation was implicated for the bobbed tail phenotype. The genomic resources for the domestic cat are now making WGS-based disease variant discovery feasible in *Felis catus,* further supporting the domestic cat as a comparative biomedical model for human studies.

## Methods

### Pedigree and clinical description

The breeding colony of Persian cats and their clinical diagnoses of PRA and bobbed tail are previously described [36, 39]. Cats were maintained under the Institutional Animal Care and Use protocols 11977, 15117 and 16691 at the University of California – Davis (UC Davis) and protocol 7808 at the University of Missouri. No additional ethical committee approval was required or requested over and above general adherence to protocols. PRA disease status was confirmed during a complete by ophthalmic examination performed by board-certified veterinary ophthalmologists from the Ophthalmology Service of the UC Davis Veterinary Medical Teaching Hospital as described [36]. All cats were over 16 weeks of age, therefore disease status was also confirmed by behavioral observations as described [36]. Tail phenotypes were confirmed by radiographs and or palpation.

**Fig. 1:**
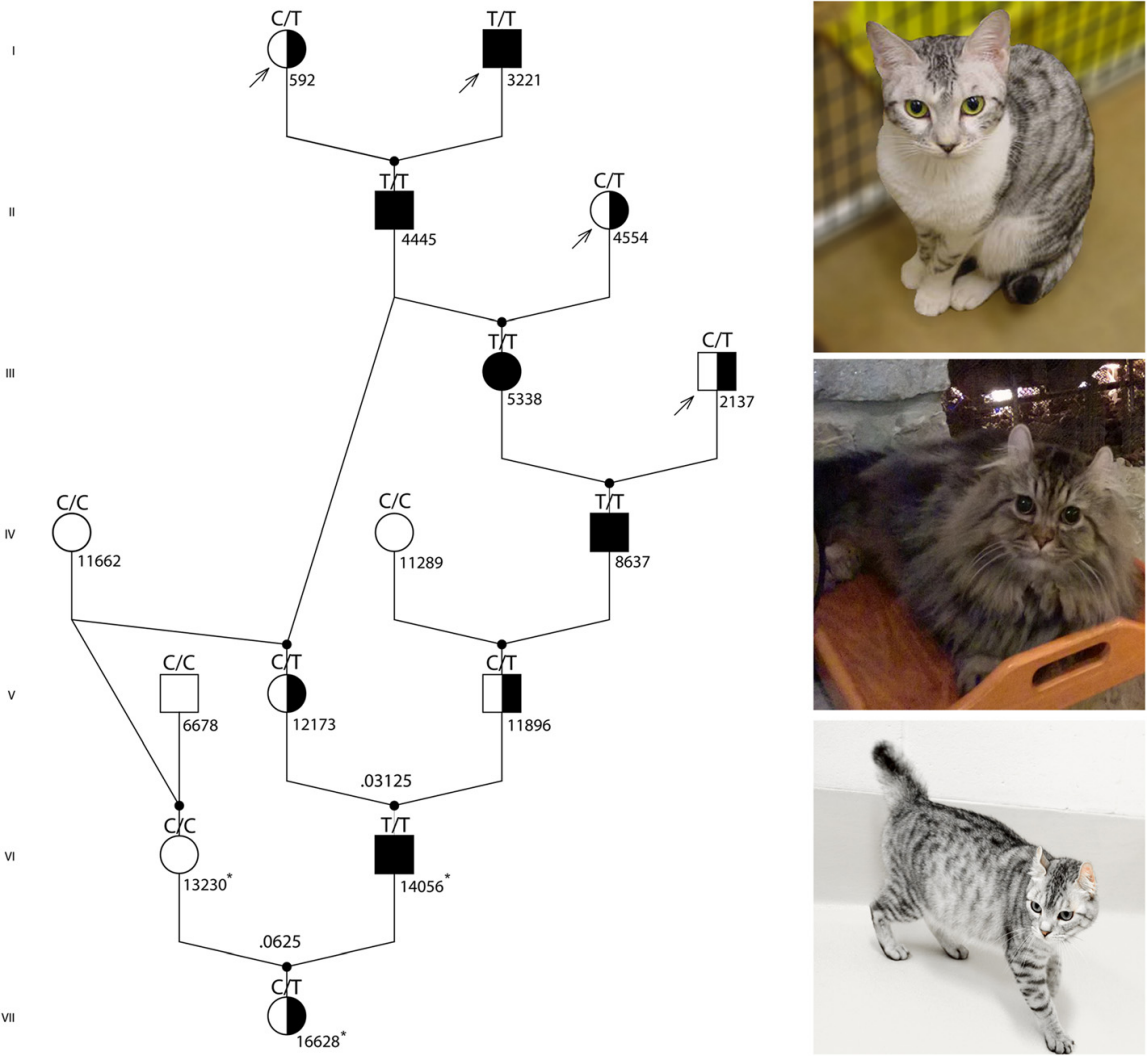
First Trio of Cats WGS for Disease Variant Identification. (*Left*) Pedigree of cats segregating for the *AIPL1* variant for Persian progressive retinal atrophy (PRA). Circles represent females, squares represent males, open symbols indicate phenotypically normal cats; solid symbols indicate Persian PRA-affected cats, half-filled symbols indicate obligate carrier cats. The arrows indicate the probands. *AIPL1* c.577C > T genotypes are indicated above each cat. Cats with an asterisk “*” are the cats used for whole genome sequencing. (*Right*) The trio of cats segregating for the Persian PRA was whole genome sequenced to potentially identify the causal variant for this animal model of LCA. Cats included (*top*) a wildtype bobbed tail queen (S13230), (middle) an PRA-affected normal tail sire (S14056), and (*bottom*) an PRA-obligate carrier female offspring (S16628) which also displays the bobbed tail. The bobbed tail trait was introduced by female cat 11662, a pedigreed cat of the Japanese Bobtail breed

Cats (*N* = 1740) from 40 different breeds, as well as random bred cats, were genotyped for the variants of interest (Additional file 1: Table S1). The majority of cats included were Persian or Persian-derived breeds (British shorthair, exotic, Himalayan, Scottish fold, Selkirk rex) [48, 49] (*n* = 1213), including 85 cats (59 sighted and 26 blind cats) from an established PRA colony [35, 36], 84 biased and 1044 unbiased Persian and Persian family samples, Table 1). A subset of these cats (*n* = 271) from 27 different breeds and random bred cats were genotyped for the *HES7* variant, including 36 colony cats (24 normal tailed and 6 bobbed-tail cats). A cat trio was selected from the larger, extended pedigree segregating as for the maximum number of traits integrated into the colony, including two forms of PRA, bobbed-tail, dorsally curled ears, hair length and six coat colors (Fig. 1). The trio included a blind sire, a non-carrier dam with a bobbed tail and a carrier offspring with a bobbed tail, Fig. 1).

**Table 1:** *AIPL1* c.577C > T Genotypes in Persian and Related Breeds

Type^a^	Population	No.	Wildtype (CC)	Carrier (CT)	Affected (TT)
Biased	Pedigree^b^	85	19	40	26
	Persian	51	43	8	0
	British Shorthair	2	2	0	0
	Exotic	29	26	3	0
	Himalayan	2	2	0	0
	Sub-total	84	73	11	0
Unbiased	Persian	707	685	22	0
	British Shorthair	111	111	0	0
	Exotic	103	102	1	0
	Himalayan	42	42	0	0
	Scottish Fold	68	67	1	0
	Selkirk Rex	13	13	0	0
	Sub-total	1044	1020	24	0
Total		1213	1112	75	0

^a^Type implies if the cat samples were biased because an owner submitted a sample specifically for the PRA genotyping or unbiased because the laboratory did a population screen of samples submitted for other genetic testing. ^b^Pedigree cats are related and not considered for allele frequency calculation. British Shorthairs, exotic, Himalayan, Scottish fold and Selkirk rex are cat breeds derived from Persians and are members of the Persian-family of cats [48]

### Cat whole genome sequencing

DNA for WGS was isolated by organic extraction from ~ 3 ml of EDTA anti-coagulated whole blood that was collected by jugular venipuncture of the trio [50]. DNA quality and quantity was visualized using ethidium bromide staining after agarose gel electrophoresis. Approximately 4 μg of high molecular weight DNA from each cat was submitted to the University of Missouri DNA Core for sequencing library preparation and NextGeneration sequencing. Sequencing libraries were constructed following the manufacturer’s protocol with reagents supplied in Illumina’s TruSeq DNA PCR-Free sample preparation kit (#FC-121-3001) (Illumina, San Diego, CA). Briefly, 1–2 μg of genomic DNA was sheared using standard Covaris (Woburn, MA) methods to prepare a 350 bp and a 550 bp library for each individual cat of the trio. The resulting 3′ and 5′ overhangs were converted to blunt ends by an end repair reaction which uses 3′ to 5′ exonuclease activity and polymerase activity. The desired size of fragment (~350 or 550 bp) was selected by sample purification beads (AMPure XP). A single adenosine nucleotide was added to the 3′ ends of the blunt fragment followed by the ligation of Illumina indexed paired-end adapters. The adaptor ligated libraries were purified twice with sample purification beads. The purified libraries then were quantified with a Qubit assay (Life Technologies, Carlsbad, CA) and library fragment size confirmed by Fragment Analyzer (Advanced Analytical Technologies, Inc., Ames, IA). Finally, the libraries were diluted and sequenced on a paired-end, 100 bp read length run according to Illumina’s standard sequencing protocol for the HiSeq 2000 (Illumina). The 350 bp and 550 bp libraries from all three cats were pooled and analyzed across nine lanes of a HiSeq 2000 (Illumina, Inc.). An average of ~30X sequencing coverage per cat was expected based on typical HiSeq output per lane and the use of PCR-free libraries.

### Genome alignment and variant calling

The demultiplexed 100 bp paired-end reads generated from the two sequencing libraries for each cat were transferred to Maverix Biomics (San Mateo, CA) for variant detection. Raw sequencing reads were checked for potential sequencing issues and contaminants using FastQC [51]. Adapter sequences, primers, Ns, and reads with quality scores below 28 were trimmed using Trimmomatic [52]. Reads with a length < 20 bp after trimming were discarded. After reassessment for quality improvement using FastQC [53], trimmed reads were mapped to the domestic cat reference genome, *Felis catus* 6.2 (http://www.ncbi.nlm.nih.gov/assembly/320798), using BWA-MEM [54]. Duplicated reads were identified and removed using SAMBLASTER [55]. Sequencing coverage across the genome for each cat was measured using DepthOfCoverage from GATK [56]. Only bases with a Phred quality score above 28 and reads with a mapping quality score above 20 were included in the coverage analysis. FreeBayes [57] was used to detect variants from the read alignments. Variants with quality scores below 20 and homopolymers were filtered from the data. The remaining variants were annotated with dbSNP ID [58] and effect predictions using SnpEff [59] based on the Ensembl gene model [60] of the reference genome. The reported variant effects and severities (e.g. high or moderate) are generated by SnpEff using variant calls, the reference genome and transcript definitions as input. Transcript definitions were obtained from the publicly available Ensembl website (www.ensembl.org). Variant effect predictions were summarized and reformatted into tab-delimited annotation files for easy interpretation and filtering. Data tracks that contained genotypes of the cat trio were prepared for visualization and data comparison in the UCSC Genome Browser [61]. Annotated variants were loaded into the variant database of the Maverix Analytic Platform that included an interactive variant exploration tool for dynamic filtering of variants based on selected effects, quality scores, gene loci, and phenotypes. To verify dynamic filtering accuracy, known phenotypic coat color variants within the trio, reviewed in Lyons [62], were verified and visually validated, including the loci for *Agouti* (*ASIP*), *Brown* (*TYRP1)*, *Dilution* (*MLPH)*, *Long* fur (*FGF5) and* ventral white *Spotting* (*KIT*).

### Genome-wide and haplotype variants analysis

A previous GWAS localized the PRA phenotype from position 713,552 - 2,076,816 on cat chromosome E1, therefore the region was scanned for disease-causing genes and variants [35]. In addition, a list of genes causing retinal diseases from the RetNet database (www.RetNet.org) was obtained and variants were visually investigated within those genes. Various studies on somite segmentation suggested *HES7* as a candidate for cat bobbed tail [40, 41, 43]. The gene is also located on cat chromosome E1, from position 2,816,901 to position 2,819,548. For the tabulations in Table 2, vcftools v0.2.12a were used to identify variants in the GWAS haplotype or in Retnet genes [63]. Segregating variants were defined as those in which the dam was homozygous for the reference allele, the sire was homozygous for the alternate allele and the offspring was heterozygote for PRA and for bobbed-tail, the dam and offspring were heterozygote for the alternative allele and the sire was homozygous for the reference allele.

**Table 2:** WGS Variants Identified in 30x Coverage of a Persian Cat PRA Trio

	Analysis type^†^			
Variant*	WGS trio	Trio Segregation	Retnet genes	GWAS haplotype
SNP	14,215,442	487,324	74,852	9607
MNP	822,020	11,856	4214	644
Insertion	1,390,990	16,656	7439	636
Deletion	1,279,028	22,134	6760	663
Mixed	304,341	3314	1588	198
Total	18,011,821	541,284	94,853	11,748

*Each alternate allele identified during variant calling is considered separately for tabulation of variant types, using the definitions at http://snpeff.sourceforge.net/SnpEff_manual.html#eff. ^†^The analyses are based on feline reference genome sequence (V6.2). The GWAS identified an associated haplotype on cat chromosome E1. For Trio Segregation, the variants segregated with the disease phenotype as well, not just parent to offspring

### *AIPL1* RNA analysis and variant genotyping

Total RNA was isolated from the retina of a sighted cat using the RNA mini kit (Invitrogen, Carlsband, CA). Complementary cDNA was synthesized as previously described (Gandolfi et al. [64]). The cDNA template was subject to PCR using 10 μM of the PCR RNA forward primer (Additional file 1: Table S2) combined with the Invitrogen polyT primer provided in the kit. The PCR conditions were: 94 °C x 4 min followed by 10 cycles conducted at 94 °C for 10 s, 66 °C for 30 s, and 68 °C for 2 min, and 20 additional cycles at 94 °C for 10 s, 62 °C for 30 s, and 68 °C for 2 min with an additional 20 s per cycle. The PCR products with appropriate lengths were purified and sequenced using four internal primers (Additional file 1: Table S2) as previously described [64].

To further correlate the suspected causative variants with the phenotypes, archival samples representing a subset of the colony cats and unrelated individuals from different breeds and random bred populations (Additional file 1: Table S1) were genotyped by direct Sanger sequencing of PCR-generated amplicons for *AIPL1* exon 4 and the *HES7* exon 1 confirmed the variants. Primer sequences are presented in Additional file 1: Table S2. PCR and thermocycling conditions were conducted as previously described with annealing at 58 °C [65]. PCR products were purified and sequenced as previously described [66]. Genotypes were also determined in broader cat populations (Table 1, Additional file 1: Table S1) by the commercial genetic typing services of the UC Davis Veterinary Genetics Laboratory using an allele-specific oligo assay and the University of Bristol, Langford Veterinary Services using a pyrosequencing-based assay (Additional file 1: Table S2). For the allele-specific assay, specificity of each primer was increased by incorporating a sequence mismatch and the addition of four mismatched bases that created a detectable product size variant (Additional file 1: Table S2). Amplicons were visualized on an ABI3730 DNA analyzer (Applied Biosystems, Foster City, CA) and analyzed using STRand software [67]. Primers for pyrosequencing were designed using PyroMark Assay Design Ver 2.0 (Qiagen, UK) (Additional file 1: Table S2). Pyrosequencing was undertaken after PCR amplification using GoTaq Master Mix (Promega, UK) of genomic DNA isolated from mouth swabs using the Nucleospin Blood kit (Macherey-Nagel, Germany) according to the manufacturer’s instructions (PyroGold, Qiagen) on a PyroMark Q24 (Qiagen). Pyrosequencing PCR was conducted using 95 °C for 2 min denaturation, followed by 38 cycles of 95 °C for 20 s, 58 °C for 40 s. For the *HES7* variant, pedigreed cats were genotyped using MALDI-TOF by submission to GeneSeek (Neogene, Inc., Lincoln, NE).

## Results

### Availability of data and materials

Sequences were submitted to the NCBI Sequence Read Archive (http://trace.ncbi.nlm.nih.gov/Traces/sra/sra.cgi?cmd=show&f=sra_sub_expl) under BioProject PRJNA288177 and included all three cats: PRA – affected, normal - tailed sire (S14056: SRS1050848), a bobbed tail dam (S13230: SRS1053073), and an obligate PRA carrier bobbed tail offspring (S16628: SRS1051523). The complete *AIPL1* CDS was obtained by RT-PCR of mRNA from the retina of a sighted cat and the sequence was submitted to the NCBI (accession #KP682504.1).

### Whole genome sequence alignment, read depth and dataset statistics

A trio of cats segregating for PRA and bobbed tail, including a PRA – affected, normal - tailed sire (S14056: SRS1050848), a bobbed tail dam (S13230: SRS1053073), and an obligate PRA carrier bobbed tail offspring (S16628: SRS1051523), was selected from the colony pedigree (Alhaddad et al. 2014) for WGS (Fig. 1). Over one billion 100 bp paired - end raw reads were obtained for each cat (1,027,975,352 in S13230, 1,100,610,902 in S14056, and 1,246,386,562 in S16628). The sequencing results were of relatively high quality with approximately 19 % of the reads removed after filtering out adapter sequences and those with low quality scores (Additional files 2 and 3: Figures S1-S2). Because no amplification was required to prepare the PCR-free libraries, an average of only 0.017 % duplicated reads among the six libraries was observed, which minimized the removal of duplicated read alignments used for variant calling and retained the maximum of read coverage. The mapping rates of the libraries to the cat reference genome were between 86.6 and 86.8 %. Average depth of coverage for each cat in the trio was 28.3X, 30X, and 34.1X with 94.3, 93.6 and 96.1 % of the genome having above 15X - coverage (Additional file 1: Table S3, Additional file 4: Figure S3).

### Variants analysis - genome-wide, candidate gene and associated haplotype

Compared to the reference, over 18,000,000 variants were detected by genome-wide sequencing the cat trio. Details for variant counts and their effects are presented in Tables 2 and 3. The number of variants that segregated concordantly with the PRA phenotype reduced to approximately 541,000 when accounting for segregation – homozygous affected sire, obligate carrier offspring and absent in the dam and reference sequence. A list of over 220 genes associated with blindness is available in the RetNet database (www.RetNet.org). Nearly 95,000 variants were found within these candidate genes and nearly 12,000 variants within the previously associated haplotype [35] (Table 3).

**Table 3:** WGS variants Identified in 30x Coverage of a Persian Cat PRA Trio

			Analysis type^b^		
Variant impact^a^	Functional Class	WGS trio	Trio Segregation	RetNet genes	GWAS haplotype
High	Stop gain	379	11	6	1
	Start/Stop loss	85	5	2	-
	Splice donor/acceptor	1262	9	9	1
	Exon deletion	2	-	-	-
	Frameshift	2254	18	20	4
	Rare amino acid	-	-	-	-
Moderate	Codon alteration	971	15	11	4
	Missense	35,444	1014	544	41
	Splice branch	-	-	-	-
	5′ or 3′ UTR Deletion	1	-	-	-
Low		64,791	2072	1155	105
Modifier		16,876,221	538,148	87,816	11,018

^a^Impact determined by snpEff as defined by http://snpeff.sourceforge.net/SnpEff_manual.html#eff. ^b^ The analyses is based on feline reference genome sequence (V6.2). Effect counts are higher than variant counts because they include the effects of each alternate allele on each nearby gene isoform. GWAS haplotype are the variants identified within the haplotype region. For Trio Segregation, the variants segregated with the disease phenotype as well, not just parent to offspring

Considering SNP effects and their impacts (Table 3), 43 high impact variants segregated concordantly with the PRA disease within the trio. Thirty-seven highly damaging variants were within the RetNet candidate genes (Table 3). The 1.3 Mb haplotype block [35], harbored six highly damaging variants, including the *AIPL1* variant, and 45 genic moderate variants (Table 3). From the phenotypes and specific genotypes of the cats, known mutations for *Agouti*, *Brown*, *Color*, *Dilution*, *Long* and *Spotting* were correctly identified by the variant calling (data not shown) [62]. The stop gain identified in *AIPL1* was considered the highest priority candidate gene mutation and was further genotyped. Approximately 101 high and moderate impact variants that segregated properly with the bobbed-tail phenotype in the trio were identified in 35 genes. The *HES7* gene was examined as an obvious candidate and a missense variant was identified in the coding region.

### Gene analysis, experimental validation and mutation genotyping

A stop gain was identified in *AIPL1,* a gene that causes LCA, at position c.577C > T of the cat coding DNA sequence. The complete *AIPL1* CDS was obtained by RT-PCR of mRNA from the retina of a sighted cat and the sequence was submitted to the NCBI (accession #KP682504.1). The complete gene coding sequence was obtained, including the complete sequence of exons 4, 5 and 6, which are not present in the current feline genome assembly V6.2. No polymorphisms were identified in the obtained sequence. The cDNA amplification generated a 987 bp long CDS and *in silico* sequence translation of feline wild-type AIPL1 generated from RNA (GenBank: KP682504) predicted a length of 329 amino acids, matching *Mus musculus* predicted AIPL1 (Additional file 5: Figure S4). The mutated *AIPL1* transcript encoded a predicted p.Arg193* protein truncation, preventing translation of approximately 40 % of the protein when compared to the wild-type sequence. When compared to human, the feline and mouse predicted AIPL1 amino acid sequences were 56 amino acids shorter, confirming that the proline-rich protein C-terminal is absent in these species. Feline DNA identity with human coding sequence (GenBank: NM_014336.4) was 76 and 80 % when compared to mouse (GenBank: XM_053245.2) while feline protein identity was 77 % when compared with both species. Feline protein sequence alignment suggested the presence of highly conserved domains when compared to the human segment: an N-terminal FK506 binding protein (FKBP-like domain) and three C-terminal tetratricopeptide repeats (TPR1, TPR2, TPR3). Homology between the feline and human FKBP, TPR1, TPR2 and TPR3 domains was 91, 94, 97 and 97 % respectively. The truncated cat protein lacked part of the TPR1 domain and the entire TPR2 and TPR3 domains.

A c.5A > G causing a p.V2A amino acid substitution was the only coding region alteration identified in *HES7*, which also segregated properly in the trio and was not present in the reference sequence. Sequence homology of cat (XM_003996191.2) and human (NM_001165967.1) is 89.9 % and protein identity is 91.4 %. The p.V2A amino acid change is prior to the defined basic helix-loop-helix and orange domains. The cat protein is also proline rich from p.128 to the C – terminus.

Cats (*N* = 1740, distributed as follows: unbiased individuals = 1558, Persian colony pedigree = 85, biased individuals = 97) from 40 different breeds, including 36 random bred cats and 61 cats with unknown origins were genotyped for the *AIPL1* variant (Table 1, Additional file 1: Table S1). A sub-set of cats (*n* = 271) was genotyped for the c.5A > G *HES7* variant. Twenty-six of 85 cats from the multi-generational pedigree and verified by ophthalmic examination to be phenotypically affected were confirmed as homozygous for the c.577C > T variant, including all three pedigree founders (Fig. 1). For 59 cats verified by ophthalmic assessment to be unaffected, 19 were homozygous wild-type and 40 heterozygous (Table 1, Additional file 1: Table S1). A majority of sighted offspring in the pedigree were expected to be heterozygous because most of the breedings were performed as test crosses of an obligate carrier to an affected mate (Fig. 1). Cats from breeds related to the Persian breed and at risk for the *AIPL1* variant included Persian, Scottish fold, Selkirk rex, British shorthair, burmilla, exotic shorthair, and Himalayan cats. No cats from a breed genetically unrelated to Persian cats had the *AIPL1* variant. No cats except those in the colony with bobbed tails (*n* = 6) and cats of the Japanese bobtail breed (*n* = 14) had the *HES7* variant. All colony cats were heterozygous for the *HES7* variant, while pedigreed Japanese bobtails were homozygotes. To estimate the variant frequency within the Persian and Persian family of breeds, 1044 cats were genotyped by two commercial service laboratories (Table 1, Additional file 1: Table S1). These samples were originally submitted for other genetic testing services. In this unbiased sampling, 24 cats were heterozygous, including 22 Persians, one Scottish fold and one exotic, suggesting an allele frequency of 1.15 %. Additionally, 84 cats were specifically submitted for PRA genotyping by cat owners, eleven were heterozygous and none were homozygous for the variant, suggesting a 6.55 % allele frequency. Because these samples were submitted by owners for PRA genotyping, the sampling is biased and the allele frequency is likely high, especially because some cats may be siblings in a litter.

## Discussion

Besides traditional rodent models, WGS has been used in cattle [68] and dogs [69–71] to identify disease mutations, however, the datasets have included hundreds of individuals for comparison. More recently, a WGS of a single cat was used to confirm a previously identified phenotypic variant for *White* [72] and a single cat compared to a variant database including 18 cats identified a feline model for Congenital Myasthenic Syndrome [47, 73]. However, the cat variant database needs to be improved for continued success, particularly for genetic studies in non-pedigreed, random bred cats [38, 74, 75]. WGS of more than one individual with an inherited disease that is identical by descent can overcome the lack of a deep variant database.

The present study represents the first comprehensive WGS effort to identify causal mutations in the domestic cat, allowing assessment of the efficiency of the WGS and variant calling process using the current cat reference assembly. In addition, other traits also segregating in this cat trio, including the bobbed tail of the Japanese bobtail breed, dorsally curled ears of the American curl, hair length, and at least six color variants were available for analysis. A targeted sequencing depth 30X genomic coverage was selected because of the number of variants that can be identified and the fraction of the genome that is callable plateaus above this depth of coverage [76]. Deep WGS of the trio of cats, including one non - PRA affected bobbed tail parent, one PRA - affected non – bobbed tail parent, and one obligate PRA carrier, bobbed tail offspring was conducted to fill sequence gaps in the targeted GWAS region for PRA in Persian cats and to support accurate variant calling. Variation was high in this trio because the family included outcrosses to several different cat breeds, including Persian, Oriental shorthair, American curl, Somali, and Bengal. Of the approximately 18 million variants identified, only 541,284 (3.0 %) had genotypes that segregated with the Persian cat PRA phenotype, 94,853 were in genes associated with vision loss (RetNet), and 11,748 that were within the GWAS haplotype region. By examining high-impact variants, stop gains were reduced from 379 overall to 11 segregating within the trio and with the phenotypes. Six stop gains were in RetNet genes and only one was in the haplotype region within an obvious LCA candidate gene, *AIPL1*. Thus, without the GWAS and a list of candidate genes, eleven stop gains would likely have been prioritized and examined over the other 32 high-impact variants segregating in the trio. Without the trio and other cat genomes for comparison, many thousands of variants could have been considered of high impact and would have required prioritization.

The DNA variant identified in the present study is a few bases upstream of a gap in the reference sequence. RNA sequencing provided the complete coding DNA sequence of *AIPL1. AIPL1* mutations affecting vision are grouped into three classes: the first class of missense mutations is located in the N-terminus, the second class of missense and stop mutations is located in the TPR motifs, and the third class of mutations includes small in-frame deletions located in the C-terminus. The first and second classes of mutations are associated with autosomal recessive LCA, while mutations in the third class appear to be associated with autosomal dominant cone-rod dystrophy and juvenile RP [34].

The abnormal vertebral presentation in the Japanese bobtail cats was an unexpected finding [39] that could be clearly associated with genes involved with somite segmentation [40–44]. The cat trio was purposely selected to segregate for a variety of traits to assist interpretation of the efficiency of the cat reference assembly and WGS efforts. The identified c.5A > G missense variant predicts a p.V2A alteration in *HES7* that appears to act dominantly to disrupt feline vertebral column development, leading to absence of either one thoracic or lumbar vertebrae and misplacement of ribs [39]. Homozygous cats have no additional abnormalities, but do have more predictable “show quality” tails. In humans, *HES7* missense changes p.R25W, p.I58V and p.D186Y cause autosomal recessive spondylocostal dysostosis (SCDO4, OMIM:613686) [45, 46].

Segregation of the *AIPL1* and *HES7* variants was confirmed in an extended pedigree of Persian cats with PRA and the introduced bobbed-tail trait. The PRA founders of the pedigree were from three geographically wide-spread locations in the USA, and from distinct lines of Persian cats. These cats were ascertained as adults in 1999 for the breeding colony, thus this form of PRA has been in the Persian population for at least 15 years. The Persian breed is the largest by population in the Cat Fanciers’ Association with over 60 % of registered cats being represented by Persians and related breeds, such as exotic shorthairs, Himalayans, Selkirk rex, Scottish fold, and British shorthairs [77]. With an allele frequency of 1.15 %, and considering an average lifespan of 12 years and a population size of at least 500,000 pedigree cats from Persian-family breeds, approximately 66 may have vision loss due to the *AIPL1* variant with approximately 11,367 carriers in the population. The *HES7* variant was private to the Japanese bobtail breed and not identified in any normal tailed breeds, however, additional bobbed tailed breeds should be examined.

The cat eye has many structural and functional similarities with the human eye, including a dual retina with rod photoreceptors that greatly outnumber cones [78]. Due to similarities between the feline and human eye, the cat has been used extensively for research involving retinal structure and visual function. Two inherited feline retinal degenerations with known causal variants are defined in Abyssinian cats. The *centrosomal protein 290* (*CEP290)* cat model [79] harbors variants also seen in approximately 30 % of humans with LCA10 [80] and the c*one rod homeobox* gene (*CRX*) is a LCA7 model [81]. The present study describes a third model, characterized by a rapidly progressive retinal dystrophy due likely to *AIPL1* deficiency. This new model should advance understanding of LCA4 pathology.

## Conclusions

The three cats reported here represent the first individuals to be sequenced as part of the 99 Lives Cat Genome Sequencing Initiative (www.felinegenetics.missouri.edu/99lives). Identified is a stop mutation in *AIPL1* of the Persian cat at position c.577C > T of the CDS producing a p.Arg193*, as predicted by the human sequence, knocking out approximately 40 % of the normal protein. Additionally, a *HES7* c.5A > G variant is associated with the bobbed tail of the Japanese bobtail breed. Cats have a longer life span than mice, affording researchers the opportunity for repeated and extended trials of gene and stem cell administration. Due to their increased longevity and larger globe relative to mice, and physiology more similar to humans, cats represent an efficient and effective model for LCA gene - and stem cell therapies.

## Additional files

Additional file 1: Table S1.
*AIPL1* c.577C > T and *HES7* c.5A > G Genotypes in Domestic Cats. **Table S2.** Primer Sequences for the Analysis of *AIPL1* in Domestic Cats. **Table S3.** Genome Coverage of Persian Cat PRA Trio. (DOCX 32 kb) Additional file 2: Figure S1. Representative Base Quality Scores of Untrimmed Sequencing Reads for the Persian cat trio. Base-specific quality scores were aggregated and plotted using FastQC (already cited, 27 and 29). Phred-scaled quality scores (y axis) are plotted against base position within the read (x axis). Box spans second and third quartiles; whiskers indicate 10th and 90th percentiles. Median and mean are plotted as red and blue lines respectively. The data aggregated here represents end X of untrimmed reads from a single library data in a single lane. (PNG 15 kb) Additional file 3: Figure S2. Sequencing Reads in the Persian Cat Trio. Quality assessment (removing low quality bases, trimming adapters and subsequently excluding reads under 20 bases) reduced the number of reads for each library and lane. Fewer reads were generated from 550 base pair libraries (those containing the text “Lib550”). The unique read counts displayed here were calculated by FastQC; duplicates reported in the text were calculated from marked duplicates among mapped reads. a) Cat 13230 non-carrier queen, b) Cat 14056 affected sire, and c) Cat 16628 carrier offspring. (ZIP 229 kb) Additional file 4: Figure S3. Bases within a Given Depth of Sequence for WGS Persian Cats. The red and green lines represent the average coverage of the parents (S13230 and S14056) and the blue line represents the average coverage of the offspring (S16628). (PNG 253 kb) Additional file 5: Figure S4. AIPL1 Protein Sequence Alignments. Presented are sequences for *Homo sapiens*, *Mus musculus, Felis silvestris catus* and *Felis silvestris catus* with the identified mutation. In green, blue, purple and grey sequences of different domains inferred using the *Homo sapiens* domain protein annotation (www.uniprot.org). In yellow, residues that differ between the *Homo sapiens* and *Mus musculus* when compared to the feline protein sequence. The *AIPL1* feline mutated sequence lacks partial TRP1 and TRP2 and TRP3. (DOCX 151 kb)

## Abbreviations

LCA: Leber’s congenital amaurosis
*AIPL1*: *aryl-hydrocarbon-interacting receptor protein-like 1*
WGS: whole genome sequencing
UC Davis: University of California – Davis
GWAS: genome-wide association study
PRA: progressive retinal atrophy
*HES7*: *Hairy And Enhancer of Split 7*
FKBP: FK506 binding protein
TPR: tetratricopeptide repeat
SNP: single nucleotide polymorphism
