# Supplementary material for: Whole genome sequencing in cats, identifies new models for blindness in AIPL1 and somite segmentation in HES7
Source: BMC Genomics. 2016 Mar 31;17:265. doi: 10.1186/s12864-016-2595-4 (PMC4815086; doi:10.1186/s12864-016-2595-4)
Supplement: Additional file 1: Table S1. — AIPL1 c.577C > T and HES7 c.5A > G Genotypes in Domestic Cats. Table S2. Primer Sequences for the Analysis of AIPL1 in Domestic Cats. Table S3. Genome Coverage of Persian Cat PRA Trio. (DOCX 32 kb) [file 12864_2016_2595_MOESM1_ESM.docx]

**Supplementary Tables**

**STable 1. *AIPL1* c.577C>T and *HES7*** **c.5A>G Genotypes in Domestic Cats**

|  |  |  | ***AIPL1* c.577C>T** | | |  | ***HES7* c.5A>G** | | |
| --- | --- | --- | --- | --- | --- | --- | --- | --- | --- |
| **Population** | **Lab** | **No.** | **Wildtype (CC)** | **Carrier (CT)** | **Affected (TT)** | **No.** | **Wildtype (AA)** | **Carrier (AG)** | **Affected (GG)** |
| **Unbiased** |  |  |  |  |  |  |  |  |  |
| Abyssinian | MU, VGL | 14 | 14 | 0 | 0 | 5 | 5 | 0 | 0 |
| American curl | VGL | 1 | 1 | 0 | 0 | 0 | 0 | 0 | 0 |
| American shorthair | MU, VGL | 15 | 15 | 0 | 0 | 8 | 8 | 0 | 0 |
| Bengal | MU, VGL | 60 | 60 | 0 | 0 | 26 | 26 | 0 | 0 |
| Birman | MU, VGL | 15 | 15 | 0 | 0 | 2 | 2 | 0 | 0 |
| British shorthair | MU, VGL | 111 | 111 | 0 | 0 | 7 | 7 | 0 | 0 |
| Burmese | MU, VGL | 8 | 8 | 0 | 0 | 13 | 13 | 0 | 0 |
| Burmilla | VGL | 3 | 3 | 0 | 0 | 0 | 0 | 0 | 0 |
| Cornish Rex | MU | 10 | 10 | 0 | 0 | 5 | 5 | 0 | 0 |
| Chartreux | MU | 8 | 8 | 0 | 0 | 3 | 3 | 0 | 0 |
| Devon Rex | MU, VGL | 13 | 13 | 0 | 0 | 5 | 5 | 0 | 0 |
| Egyptian Mau | MU | 7 | 7 | 0 | 0 | 4 | 4 | 0 | 0 |
| Exotic shorthair | MU, VGL | 103 | 102 | 1 | 0 | 0 | 0 | 0 | 0 |
| Highlander | VGL | 2 | 2 | 0 | 0 | 0 | 0 | 0 | 0 |
| Himalayan | VGL | 42 | 42 | 0 | 0 | 0 | 0 | 0 | 0 |
| Japanese Bobtail | MU | 8 | 8 | 0 | 0 | 14 | 0 | 0 | 14 |
| Korat | MU | 7 | 7 | 0 | 0 | 0 | 0 | 0 | 0 |
| La Perm | MU | 0 | 0 | 0 | 0 | 46 | 46 | 0 | 0 |
| Maine Coon | MU, VGL | 47 | 47 | 0 | 0 | 7 | 7 | 0 | 0 |
| Manx | MU | 7 | 7 | 0 | 0 | 5 | 5 | 0 | 0 |
| Napoleon | VGL | 5 | 5 | 0 | 0 | 0 | 0 | 0 | 0 |
| Norwegian Forest Cat | MU, VGL | 10 | 10 | 0 | 0 | 7 | 7 | 0 | 0 |
| Ocicat | MU | 7 | 7 | 0 | 0 | 4 | 4 | 0 | 0 |
| Oriental | MU, VGL | 8 | 8 | 0 | 0 | 26 | 26 | 0 | 0 |
| Persian | MU, VGL, LVS | 707 | 685 | 22 | 0 | 3 | 3 | 0 | 0 |
| Ragamuffin | VGL | 5 | 5 | 0 | 0 | 0 | 0 | 0 | 0 |
| Ragdoll | MU, VGL | 76 | 76 | 0 | 0 | 3 | 3 | 0 | 0 |
| Russian Blue | MU | 8 | 8 | 0 | 0 | 2 | 2 | 0 | 0 |
| Savannah cat | VGL | 3 | 3 | 0 | 0 | 0 | 0 | 0 | 0 |
| Scottish Fold | MU, VGL | 68 | 67 | 1 | 0 | 0 | 0 | 0 | 0 |
| Selkirk Rex | MU, VGL | 13 | 13 | 0 | 0 | 0 | 0 | 0 | 0 |
| Siamese | MU, VGL | 12 | 12 | 0 | 0 | 5 | 5 | 0 | 0 |
| Siberian | MU, VGL | 23 | 23 | 0 | 0 | 5 | 5 | 0 | 0 |
| Somali | VGL | 2 | 2 | 0 | 0 | 0 | 0 | 0 | 0 |
| Sphynx | MU, VGL | 11 | 11 | 0 | 0 | 4 | 4 | 0 | 0 |
| Tonkinese | MU | 7 | 7 | 0 | 0 | 6 | 6 | 0 | 0 |
| Toyger | VGL | 4 | 4 | 0 | 0 | 0 | 0 | 0 | 0 |
| Turkish Angora | MU, VGL | 7 | 7 | 0 | 0 | 5 | 5 | 0 | 0 |
| Turkish Van | MU, VGL | 4 | 4 | 0 | 0 | 4 | 4 | 0 | 0 |
| Random Bred | MU, VGL | 36 | 36 | 0 | 0 | 10 | 10 | 0 | 0 |
| Unknown | VGL | 61 | 61 | 0 | 0 | 7 | 7 | 0 | 0 |
| Sub-total (39 Breeds) |  | 1558 | 1534 | 24 | 0 | 241 | 227 | 0 | 14 |
| **Biased** |  |  |  |  |  |  |  |  |  |
| Colony | MU | 85 | 19 | 40 | 26 | 30 | 24 | 6 | 0 |
| Abyssinian | VGL | 3 | 3 | 0 | 0 | 0 | 0 | 0 | 0 |
| Australian Mist | VGL | 3 | 3 | 0 | 0 | 0 | 0 | 0 | 0 |
| Bengal | VGL | 2 | 2 | 0 | 0 | 0 | 0 | 0 | 0 |
| Birman | VGL | 1 | 1 | 0 | 0 | 0 | 0 | 0 | 0 |
| British shorthair | LVS | 2 | 2 | 0 | 0 | 0 | 0 | 0 | 0 |
| European shorthair | VGL | 2 | 2 | 0 | 0 | 0 | 0 | 0 | 0 |
| Exotic shorthair | VGL, LVS | 29 | 26 | 3 | 0 | 0 | 0 | 0 | 0 |
| Himalayan | LVS | 2 | 2 | 0 | 0 | 0 | 0 | 0 | 0 |
| Persian | VGL, LVS | 51 | 43 | 8 | 0 | 0 | 0 | 0 | 0 |
| Seychelle | LVS | 1 | 1 | 0 | 0 | 0 | 0 | 0 | 0 |
| Savannah | LVS | 1 | 1 | 0 | 0 | 0 | 0 | 0 | 0 |
| Sub-total (11 breeds) |  | 97 | 86 | 11 | 0 | 0 | 0 | 0 | 0 |
| **Totals (41 breeds)** |  | **1740** | **1639** | **75** | **26** | **271** | **251** | **6** | **14** |

*Type implies if the cats were related from the pedigree, or biased because a breeder submitted a sample specifically for the PRA genotyping or unbiased because the laboratory did a population screen of samples submitted for other genetic testing. ^†^These Persian cats represent two unrelated cats and a sibship of four kittens. All colony cats were F1 offspring of breedings from a pedigreed Japanese bobtail (homozygote) or F1 cats bred with a normal tailed cat.

**STable 2. Primer Sequences for the Analysis of *AIPL1* in Domestic Cats.**

| **Assay** | **5’ Primer (5’-3’)** | **3’ Primer (5’-3’)** | **Seq primer (5’-3’)** |
| --- | --- | --- | --- |
| PCR *AIPL1* RNA | caactactgccagtgtctgctgaag | Invitrogen Poly-T |  |
| *AIPL1* |  |  | ctcaacattgtcctgaccccc |
| RNA-Sequencing |  |  | caacaccctgatcctcaactactgc |
|  |  |  | caacaccctgatcctcaactactgc |
|  |  |  | caactactgccagtgtctgctgaag |
| *AIPL1* genotyping | gtgttgacaaagggcggtcttt | ggttccgcaggcacacga |  |
| *HES7* genotyping | ccaacttttatttgagggtcggt | agccccccttatttattccactc |  |
| Sequenom assay  *HES7* | tgcgggatataaggtgcaag | cctattctccgctcgatcc |  |
| Allele specific assay  *AIPL1* | gacgtggagcctgagtaacg  ggaggcctcctcgaatcg | gttcggaggcctcctcgtttca |  |
| Pyrosequencing | aagggaaccggctcttca | Biotin-ttccgcaggcacacgat | cggctcttcaagctgggc |

*Primers used for RNA internal sequencing.

**STable 3.** **Genome Coverage of Persian Cat PRA Trio.**

|  |  | **Percentage of genome coverage** | | | | | |
| --- | --- | --- | --- | --- | --- | --- | --- |
| **Sample** | **Mean Depth** | **> 10X** | **> 15X** | **> 20X** | **> 25X** | **> 30X** | **> 35X** |
| S13230 | 28.29 | 98.1 | 94.3 | 84.3 | 66.1 | 43.3 | 22.8 |
| S14056 | 30.00 | 97.8 | 93.8 | 85.3 | 70.8 | 51.5 | 31.4 |
| S16628 | 34.13 | 98.4 | 96.1 | 91.0 | 81.7 | 67.1 | 48.4 |
