# Supplementary figures and images for: Whole genome sequencing in cats, identifies new models for blindness in AIPL1 and somite segmentation in HES7
Source: BMC Genomics. 2016 Mar 31;17:265. doi: 10.1186/s12864-016-2595-4 (PMC4815086; doi:10.1186/s12864-016-2595-4)

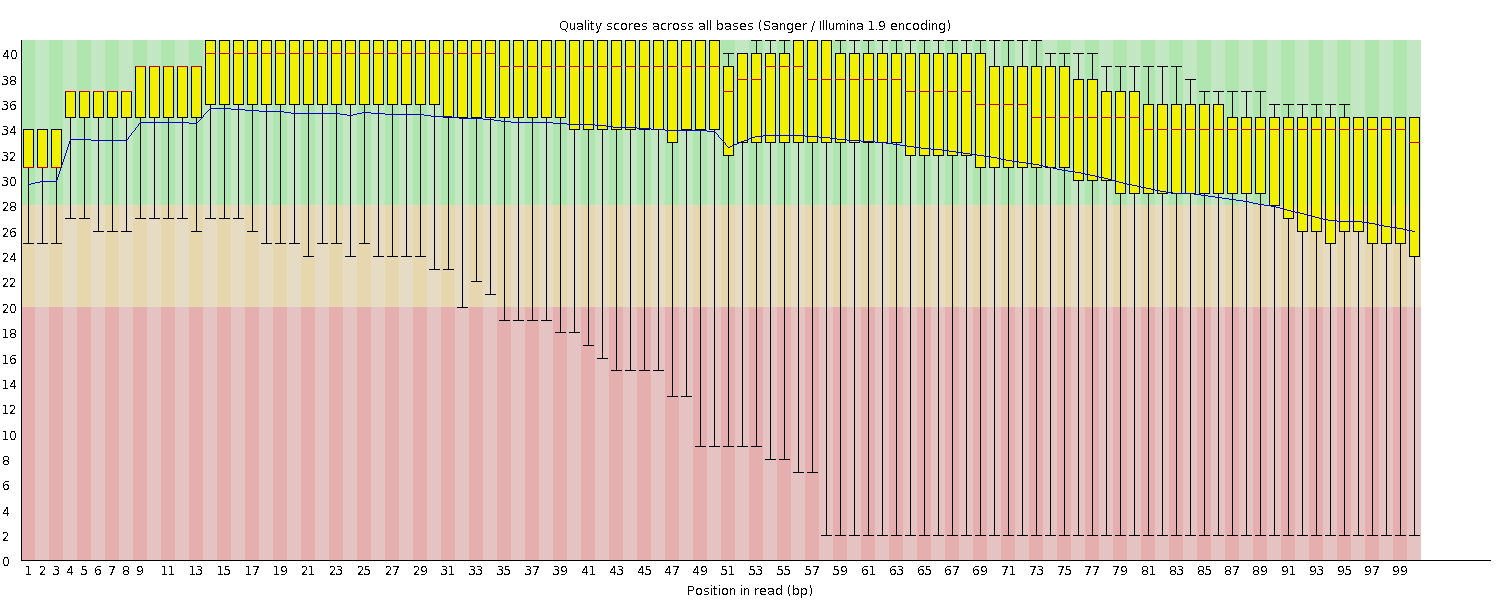

Supplement: Additional file 2: Figure S1. — Representative Base Quality Scores of Untrimmed Sequencing Reads for the Persian cat trio. Base-specific quality scores were aggregated and plotted using FastQC (already cited, 27 and 29). Phred-scaled quality scores (y axis) are plotted against base position within the read (x axis). Box spans second and third quartiles; whiskers indicate 10th and 90th percentiles. Median and mean are plotted as red and blue lines respectively. The data aggregated here represents end X of untrimmed reads from a single library data in a single lane. (PNG 15 kb) [file 12864_2016_2595_MOESM2_ESM.png]

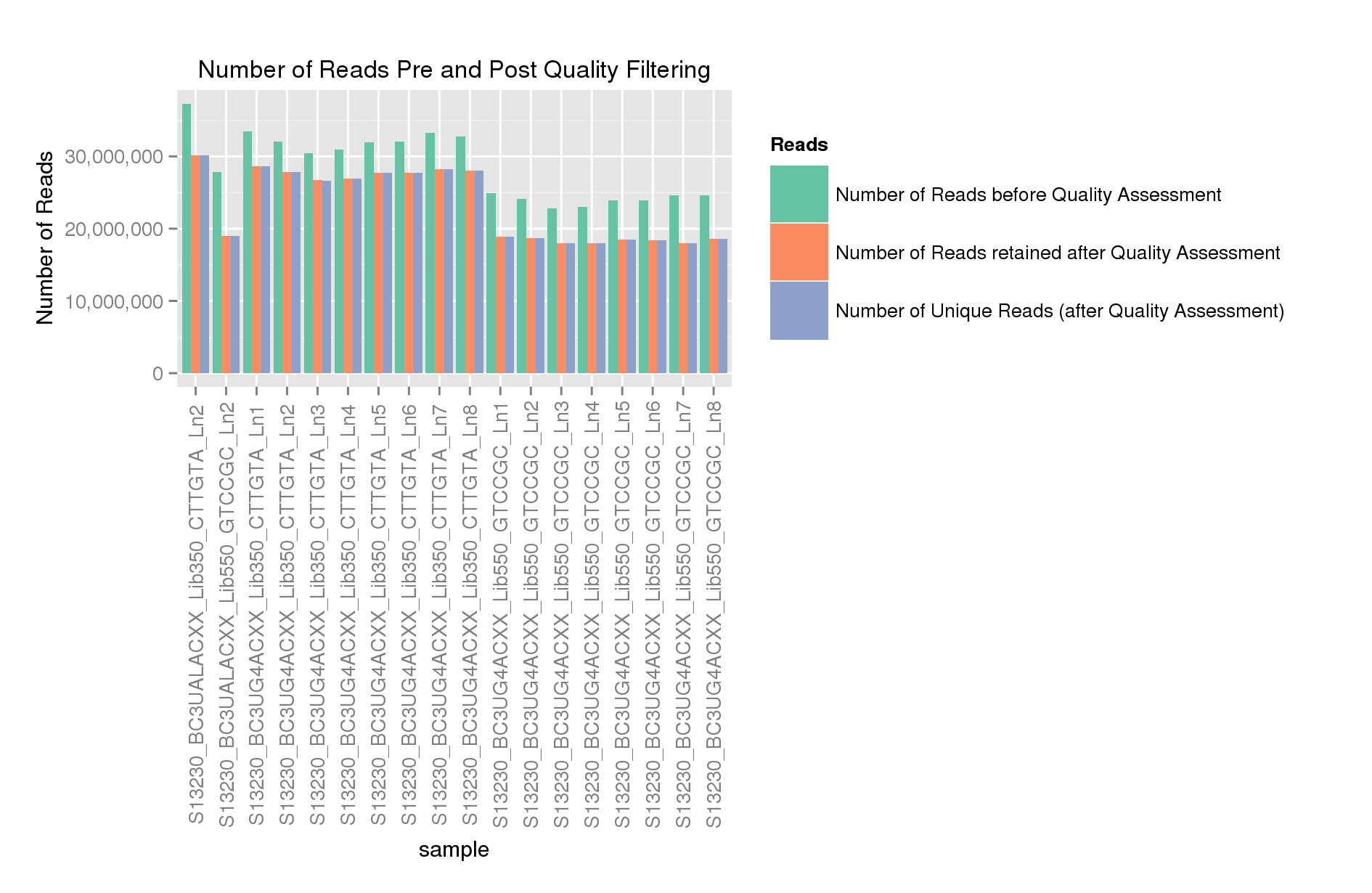

Supplement: Additional file 3: Figure S2. — Sequencing Reads in the Persian Cat Trio. Quality assessment (removing low quality bases, trimming adapters and subsequently excluding reads under 20 bases) reduced the number of reads for each library and lane. Fewer reads were generated from 550 base pair libraries (those containing the text “Lib550”). The unique read counts displayed here were calculated by FastQC; duplicates reported in the text were calculated from marked duplicates among mapped reads. a) Cat 13230 non-carrier queen, b) Cat 14056 affected sire, and c) Cat 16628 carrier offspring. (ZIP 229 kb) [file 12864_2016_2595_MOESM3_ESM.zip › sfig2/PPRA_WGS_SFig2a.png]

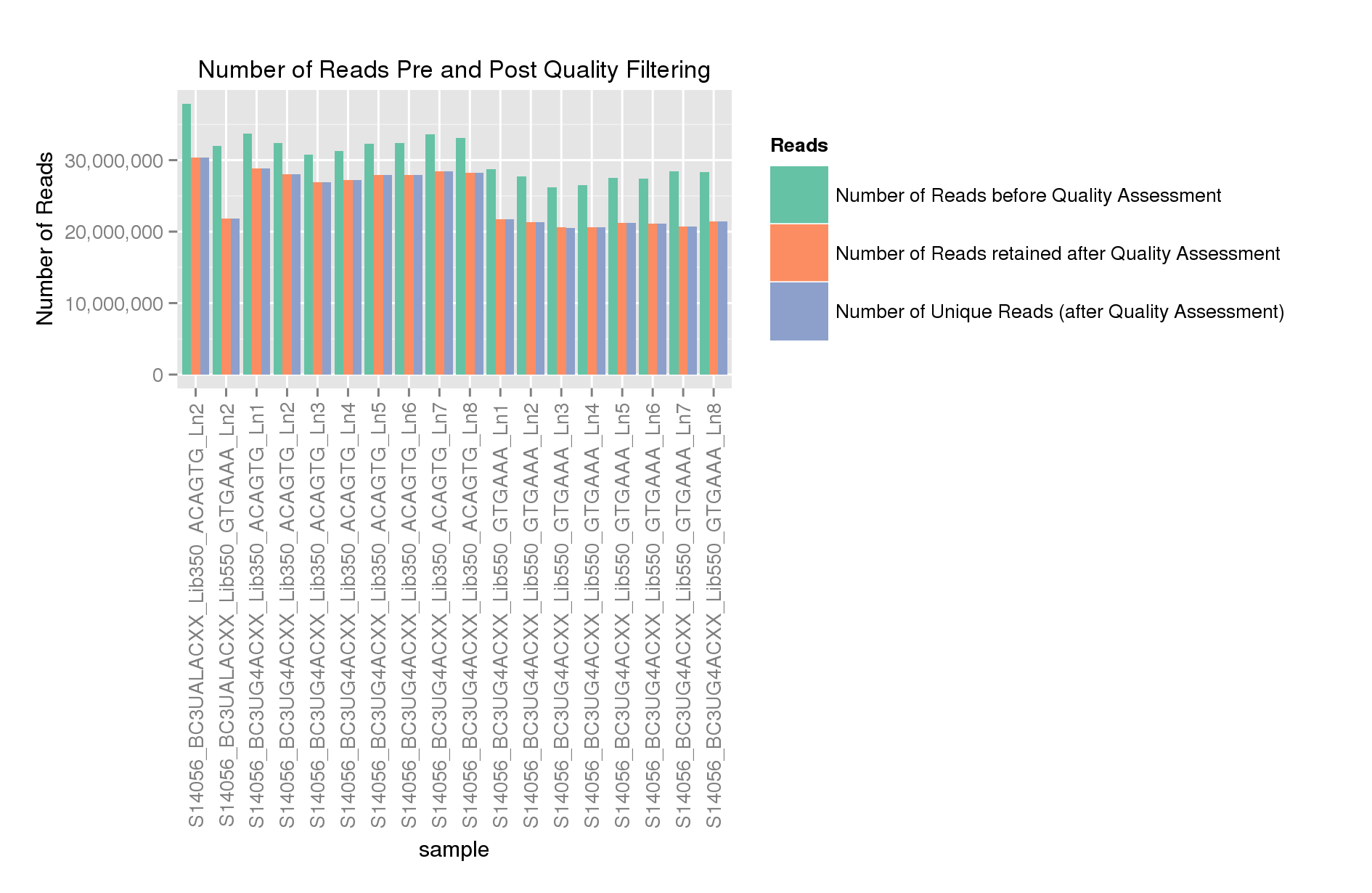

Supplement: Additional file 3: Figure S2. — Sequencing Reads in the Persian Cat Trio. Quality assessment (removing low quality bases, trimming adapters and subsequently excluding reads under 20 bases) reduced the number of reads for each library and lane. Fewer reads were generated from 550 base pair libraries (those containing the text “Lib550”). The unique read counts displayed here were calculated by FastQC; duplicates reported in the text were calculated from marked duplicates among mapped reads. a) Cat 13230 non-carrier queen, b) Cat 14056 affected sire, and c) Cat 16628 carrier offspring. (ZIP 229 kb) [file 12864_2016_2595_MOESM3_ESM.zip › sfig2/PPRA_WGS_SFig2b.png]

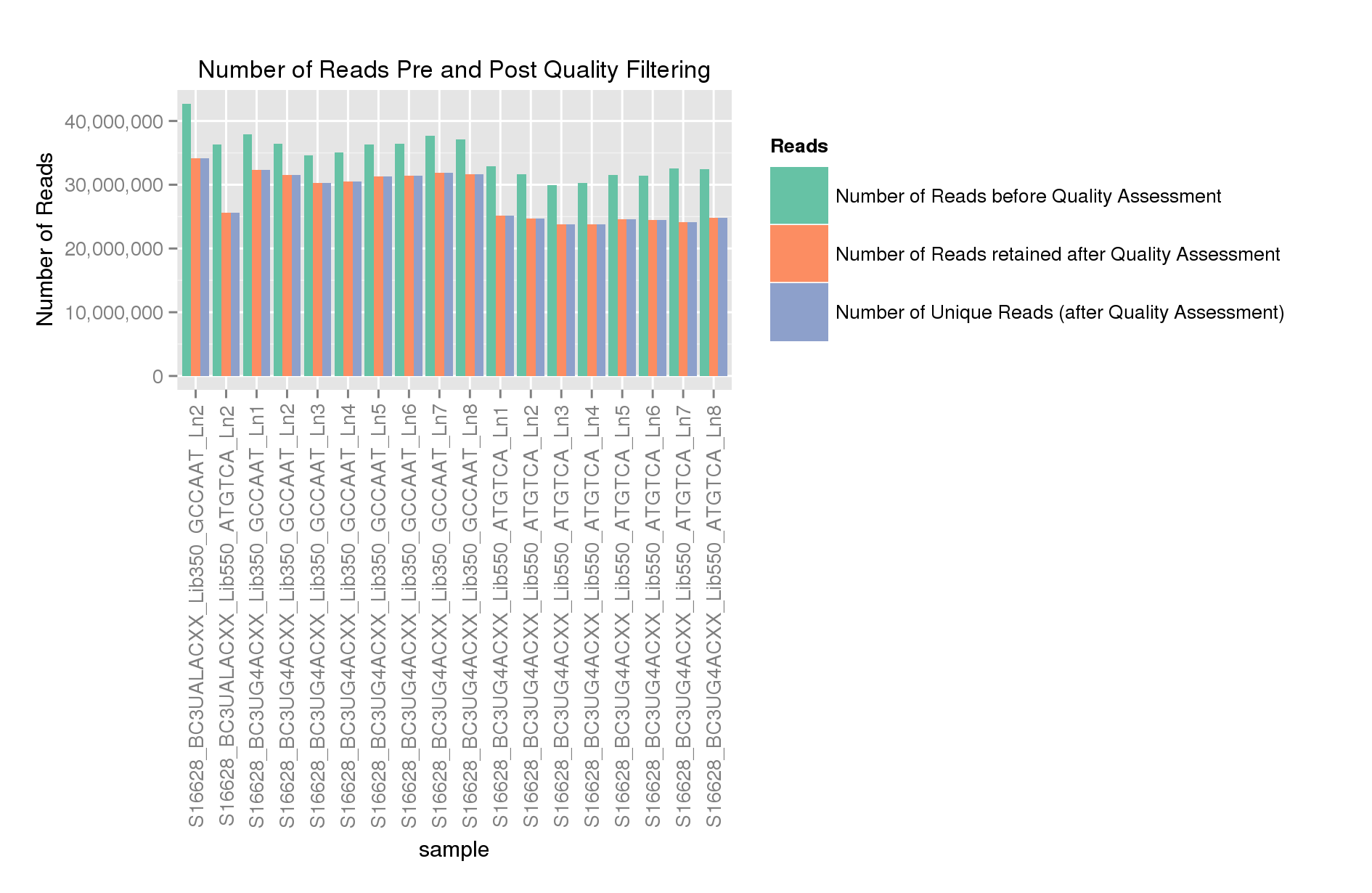

Supplement: Additional file 3: Figure S2. — Sequencing Reads in the Persian Cat Trio. Quality assessment (removing low quality bases, trimming adapters and subsequently excluding reads under 20 bases) reduced the number of reads for each library and lane. Fewer reads were generated from 550 base pair libraries (those containing the text “Lib550”). The unique read counts displayed here were calculated by FastQC; duplicates reported in the text were calculated from marked duplicates among mapped reads. a) Cat 13230 non-carrier queen, b) Cat 14056 affected sire, and c) Cat 16628 carrier offspring. (ZIP 229 kb) [file 12864_2016_2595_MOESM3_ESM.zip › sfig2/PPRA_WGS_SFig2c.png]

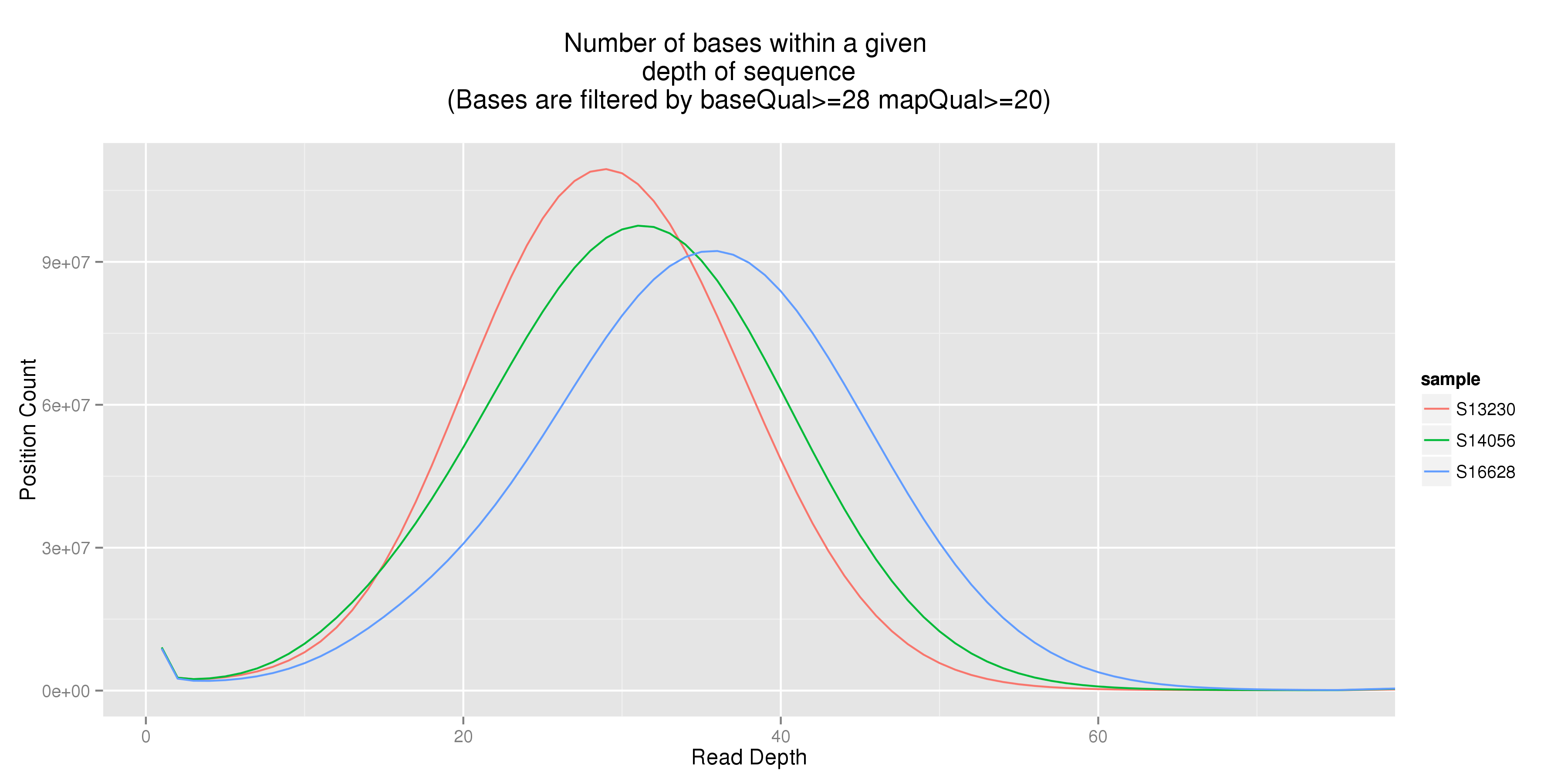

Supplement: Additional file 4: Figure S3. — Bases within a Given Depth of Sequence for WGS Persian Cats. The red and green lines represent the average coverage of the parents (S13230 and S14056) and the blue line represents the average coverage of the offspring (S16628). (PNG 253 kb) [file 12864_2016_2595_MOESM4_ESM.png]
