# Supplementary material for: Whole genome sequencing in cats, identifies new models for blindness in AIPL1 and somite segmentation in HES7
Source: BMC Genomics. 2016 Mar 31;17:265. doi: 10.1186/s12864-016-2595-4 (PMC4815086; doi:10.1186/s12864-016-2595-4)
Supplement: Additional file 5: Figure S4. — AIPL1 Protein Sequence Alignments. Presented are sequences for Homo sapiens, Mus musculus, Felis silvestris catus and Felis silvestris catus with the identified mutation. In green, blue, purple and grey sequences of different domains inferred using the Homo sapiens domain protein annotation (www.uniprot.org). In yellow, residues that differ between the Homo sapiens and Mus musculus when compared to the feline protein sequence. The AIPL1 feline mutated sequence lacks partial TRP1 and TRP2 and TRP3. (DOCX 151 kb) [file 12864_2016_2595_MOESM5_ESM.docx]

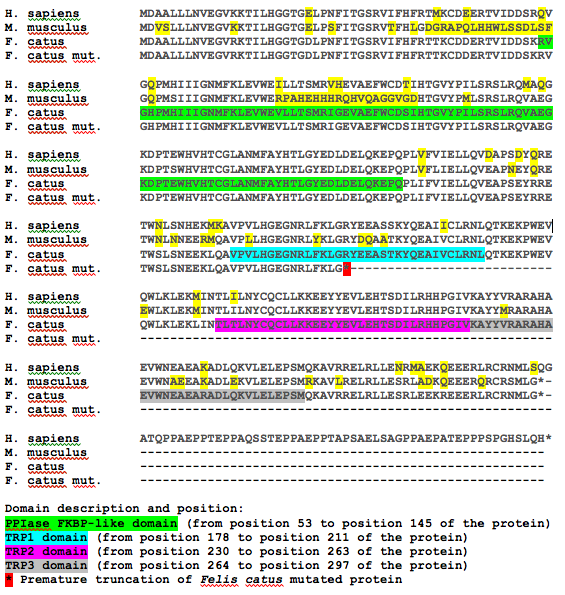


**Supplementary Figure 4**. AIPL1 protein sequence alignments in *Homo sapiens*, *Mus musculus, Felis catus* and *Felis catus* with the identified mutation. In green, blue, purple and grey sequences of different domains inferred using the *Homo sapiens* domain protein annotation ([www.uniprot.org](http://www.uniprot.org)). In yellow, residues that differ between the *Homo sapiens* and *Mus musculus* when compared to the feline protein sequence. The *AIPL1* feline mutated sequence lacks partial TRP1 and TRP2 and TRP3.
